# A Novel Role for Pro-Coagulant Microvesicles in the Early Host Defense against *Streptococcus pyogenes*

**DOI:** 10.1371/journal.ppat.1003529

**Published:** 2013-08-01

**Authors:** Sonja Oehmcke, Johannes Westman, Johan Malmström, Matthias Mörgelin, Anders I. Olin, Bernd Kreikemeyer, Heiko Herwald

**Affiliations:** 1 University Medicine, Institute of Medical Microbiology, Virology and Hygiene, Rostock University, Rostock, Germany; 2 Department of Clinical Sciences, Division of Infection Medicine, Lund University, Lund, Sweden; 3 Department of Immunotechnology, Lund University, Lund, Sweden; National Institute of Allergy and Infectious Diseases, National Institutes of Health, United States of America

## Abstract

Previous studies have shown that stimulation of whole blood or peripheral blood mononuclear cells with bacterial virulence factors results in the sequestration of pro-coagulant microvesicles (MVs). These particles explore their clotting activity via the extrinsic and intrinsic pathway of coagulation; however, their pathophysiological role in infectious diseases remains enigmatic. Here we describe that the interaction of pro-coagulant MVs with bacteria of the species *Streptococcus pyogenes* is part of the early immune response to the invading pathogen. As shown by negative staining electron microscopy and clotting assays, pro-coagulant MVs bind in the presence of plasma to the bacterial surface. Fibrinogen was identified as a linker that, through binding to the M1 protein of *S. pyogenes*, allows the opsonization of the bacteria by MVs. Surface plasmon resonance analysis revealed a strong interaction between pro-coagulant MVs and fibrinogen with a K_D_ value in the nanomolar range. When performing a mass-spectrometry-based strategy to determine the protein quantity, a significant up-regulation of the fibrinogen-binding integrins CD18 and CD11b on pro-coagulant MVs was recorded. Finally we show that plasma clots induced by pro-coagulant MVs are able to prevent bacterial dissemination and possess antimicrobial activity. These findings were confirmed by *in vivo* experiments, as local treatment with pro-coagulant MVs dampens bacterial spreading to other organs and improved survival in an invasive streptococcal mouse model of infection. Taken together, our data implicate that pro-coagulant MVs play an important role in the early response of the innate immune system in infectious diseases.

## Author Summary

The coagulation system is much more than a passive bystander in our defense against exogenous microorganisms. Over the last years there has been a growing body of evidence pointing to an integral part of coagulation in innate immunity and a special focus has been on bacterial entrapment in a fibrin network. However, thus far, pro-coagulant MVs have not been discussed in this context, though it is known that their numbers can dramatically increase in many pathological conditions, including severe infectious diseases. In the present study we see a significant increase of pro-coagulant MVs in an invasive streptococcal mouse model, suggesting that their release is an immune response to the infection. We find that pro-coagulant MVs bind to *Streptococcus pyogenes* and promote clotting, entrapment, and killing of the bacteria in a fibrin network. As a proof of concept pro-coagulant MVs were applied as local treatment in the streptococcal infection model, and it was demonstrated that this led to a significantly improved survival in mice.

## Introduction

Today it is generally accepted that coagulation is tightly interwoven with the innate immune system [1]. Both systems can act in a combined effort to sense and eradicate an infection in a highly sophisticated manner. Indeed, evolutionary studies suggest that fibrinogen has relatively recently acquired its function as a clotting factor because many fibrinogen-related proteins in invertebrates have an important role in defense processes, such as pathogen recognition, agglutination, and bacterial lysis, however, not in clotting [2]. This applies also to other members of the coagulation cascade, as sequence homology analyses in vertebrates revealed that many clotting factors share ancestry with complement proteases [3]. Together these results show that the vertebrate coagulation system has developed from evolutionary related cascades involved in innate immunity [4]. It is therefore tempting to speculate that coagulation has a yet underestimated function in the host defense to infection. The coagulation cascade can be broken down into an extrinsic (tissue factor driven) and intrinsic pathway (contact activation). Both arms are initiated by limited proteolysis and are amplified in a snowball-like manner, eventually resulting in the generation of thrombin, which then initiates formation of a fibrin network [5].

The Gram-positive bacterium *Streptococcus pyogenes* is a major human pathogen that mainly causes local and self-limiting skin and throat infections. Infections can occasionally become invasive and develop into serious and life-threatening conditions such as streptococcal toxic shock syndrome (STSS) and necrotizing fasciitis. Notably, both conditions are associated with high morbidity and mortality (for a review see [6]). The bacterium has evolved a variety of strategies to evoke activation of the coagulation cascade, involving for instance the induction of tissue factor on monocytes and endothelial cells by M proteins or an activation of the intrinsic pathway at the bacterial surface [7]–[9]. M proteins are streptococcal surface proteins and probably one of the best-known virulence determinants of this pathogen [10]. They can be released during infections [11] and act on monocytes to trigger cytokine induction and tissue factor up-regulation [8], [12]. Recently we reported that soluble M protein triggers the release of pro-coagulant MVs from human peripheral blood mononuclear cells (PBMCs). Once released from PBMCs these MVs can initiate coagulation by activating both pathways in a sequential mode of action [13].

Apart from PBMCs MVs can be secreted from almost all other human blood-born cells, and depending on their cell activation MVs can differ in their composition and function. Elevated levels of MVs have been related to pathological conditions such as bleeding and thrombotic disorders, cardiovascular diseases, cancer, and infectious diseases [14]. They form sphere-shaped structures, less than 1 µm of diameter and limited by a lipid bilayer. In contrast to their cell of origin, MVs from activated cells expose negatively charged phospholipids, mainly phosphatidylserine (PS), on their outer membrane, which present a neo-exposed docking site for many plasma proteins including coagulation factors [15].

Despite an increasing knowledge on the role(s) of MVs in pathological processes e.g. as signaling molecules, in angiogenesis, and in initiation or propagation of coagulation and inflammation [14], their function in infectious diseases is only poorly understood. In the present study we investigated whether pro-coagulant MVs are part of the innate immune response by exposing antimicrobial activity. To this end we performed a number of *in vitro* and *in vivo* experiments to show that pro-coagulant MVs not only efficiently prevent the proliferation of *S. pyogenes* bacteria within a formed clot, but also that application of human MVs in a subcutaneous murine infection model dampens bacterial spreading and improves survival.

## Results

### Pro-coagulant MVs bind to *S. pyogenes*

**Figure 1:**
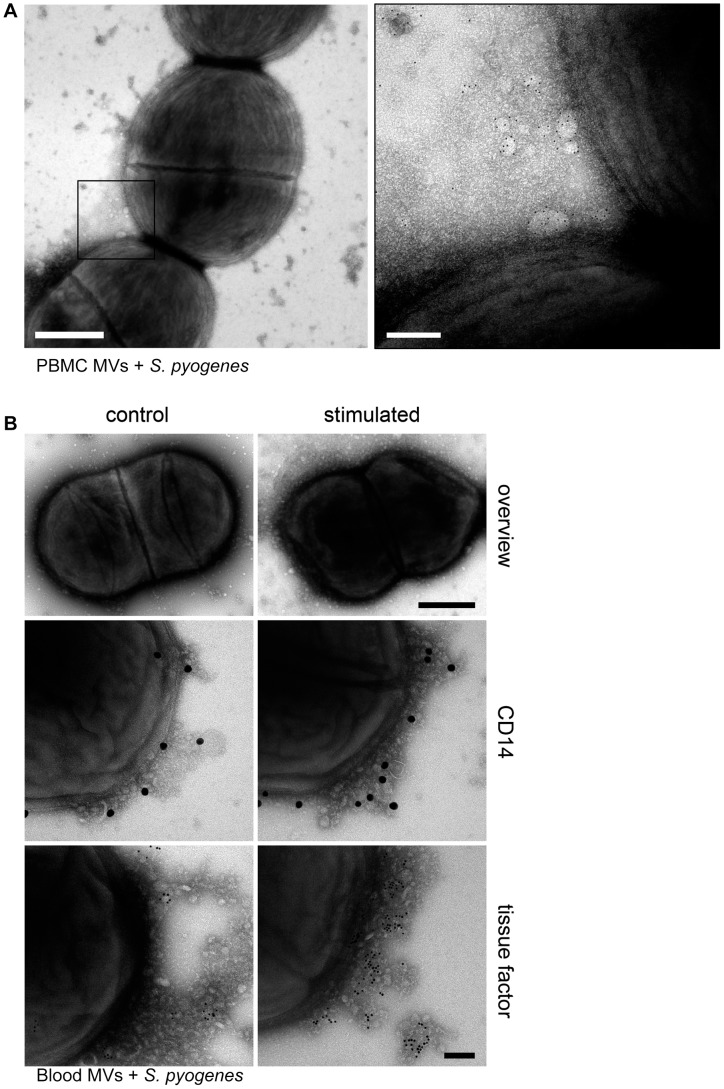
MVs bind to *S.* *pyogenes*. **A**) Pro-coagulant MVs were gold-labeled with annexin V (5 nm) and incubated with *S. pyogenes* bacteria in the presence of 1% plasma. Samples were processed by negative staining and analyzed in a transmission electron microscope at two different magnifications. Arrows point to annexin V-positive MVs and scale bars represent 500 nm (left) or 100 nm (right). **B**) An overview of an *S. pyogenes* bacterium opsonized with ctrl. (*upper panel left site*) and pro-coagulant MVs (*upper panel right site*) is shown (Scale bar 500 nm). Ctrl. MVs (*left side*) and pro-coagulant MVs (*right side*) were immunostained with a gold-labeled antibody against CD14 (*middle panel*) or tissue factor (*lower panel*) and incubated with *S. pyogenes*. The scale bar indicates 100 nm.

PBMCs were isolated from human blood and stimulated with M1 protein as described in *Methods*. MVs were then purified as reported earlier [13] and the pro-coagulant activity of MVs was confirmed by measuring the clotting time (data not shown). For subsequent binding studies, pro-coagulant MVs were tagged with gold-labeled annexin V and incubated with *S. pyogenes* bacteria in the presence of 1% plasma. Figure 1A depicts transmission electron micrographs at lower and higher magnification. At higher magnification the figure shows that pro-coagulant MVs are bound to the bacterial surface in the presence of plasma.

To test whether the presence of MVs derived from other cells, interferes with the binding of pro-coagulant MVs from PBMCs, whole blood was stimulated with M1 protein. MVs were isolated and their binding to *S. pyogenes* was studied by transmission electron microscopy. Figure 1B (*upper panel*) shows that MVs isolated from M1 protein-activated blood bind to the bacterial surface. The origin of PBMC-derived MVs was confirmed by immunostaining with CD14, also showing that activation of blood with M1 protein caused an increase in binding of monocyte-derived MVs (Figure 1B, *middle panel*). To test whether the activation stage of the MVs contributes to binding, MVs were immunostained with an antibody against tissue factor. Figure 1B (*lower panel*) shows that only a few tissue factor-positive MVs were found attached to the bacteria, when MVs were isolated from non-stimulated blood. However, a more intensive antibody staining was recorded when MVs were recovered from M1 protein stimulated blood, showing that blood cell activation led to pro-coagulant MVs that bind to the bacterial surface. Based on these results we decided to use MVs isolated from PBMCs for all further experiments. MVs that were isolated from M1 protein stimulated PBMCs are therefore referred to as “pro-coagulant MVs” and from non-activated PBMCs as “ctrl. MVs” throughout the remaining part of this study.

The interaction of MVs with *S. pyogenes* was further investigated by fluorescence microscopy. Pro-coagulant or ctrl. MVs were labeled with PKH26 (red) and incubated with *S. pyogenes* in human plasma. After a 30 minute incubation step, aggregates of MVs and bacteria (DAPI-stained, blue) were observed (Figure S1), similar to those described by Timár and colleagues [16]. The number of MV-bacterial aggregates that exceeded 10 µm was quantified (Table 1). The data show that both types of MVs bind and aggregate bacteria, but incubation with pro-coagulant MVs induced more and larger aggregates when compared with ctrl-MVs (Table 1).

**Table 1:** Number of MV-bacteria aggregates (≥10 µm sized).

*S. pyogenes* + ctrl. MVs	*S. pyogenes* + pro-coagulant MVs
19±4	49±11

PKH-26 stained MVs and *S. pyogenes* were incubated in plasma for 30 min. 10 µl was dropped onto a cover slide, and counterstained with DAPI. Statistical analysis was carried out from 35 microscopic visual areas. Average and SEM are shown from 3 independent experiments.

### Clotting of *S. pyogenes* after opsonization with pro-coagulant MVs

**Figure 2:**
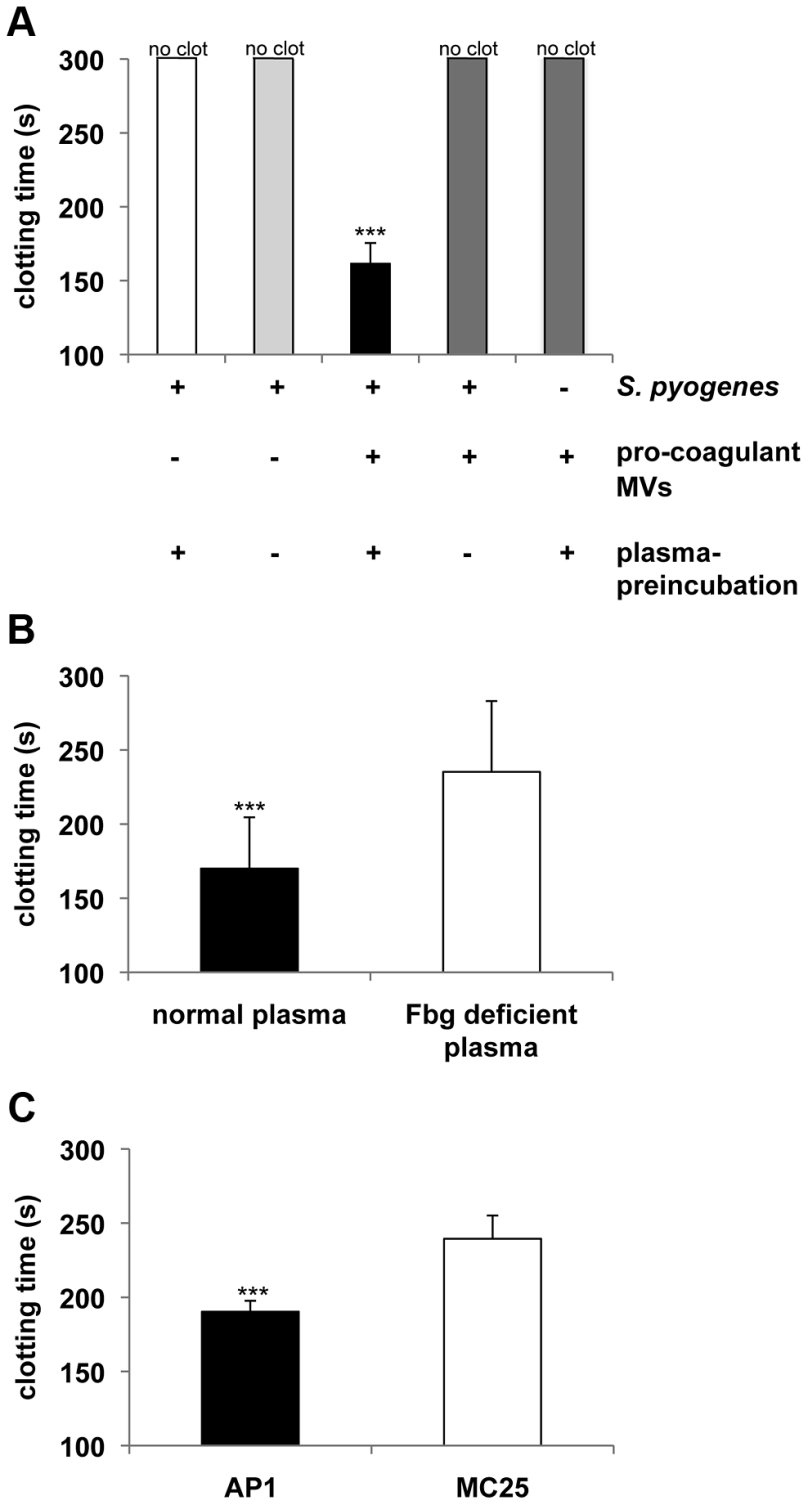
Pro-coagulant MVs bound to *S.* *pyogenes* induce clotting in human plasma. **A**) *S. pyogenes* were incubated with pro-coagulant MVs or buffer in the presence or absence of plasma for 30 min at 37°C. After washing, bacteria were added to recalcified plasma and clotting time was determined. **B**) *S. pyogenes* were incubated with pro-coagulant MVs in the presence of normal or fibrinogen (Fbg) depleted plasma for 30 min at 37°C. After washing, bacteria were added to recalcified plasma and clotting time was determined. **C**) Wildtype AP1 or MC25 bacteria were incubated with pro-coagulant MVs in the presence of plasma for 30 min at 37°C. After washing, bacteria were added to recalcified plasma and clotting time was determined. Clotting times were performed in triplicate. The data represent the means ± SD of 3 independent experiments. ***P<0.001.

Next we tested whether opsonization of *S. pyogenes* with pro-coagulant MVs, renders the bacteria susceptible for clotting. To this end, *S. pyogenes* bacteria were pre-incubated with pro-coagulant MVs in the presence or absence of human plasma, washed thoroughly to remove non-bound MVs, and added to recalcified plasma. Under these experimental settings clotting occurred within 162 s as shown in figure 2A. If, however, bacteria were incubated with pro-coagulant MVs in the absence of human plasma, no clotting was observed within 300 s and likewise, incubation of bacteria with plasma in the absence of pro-coagulant MVs prevented clotting (Figure 2A). Together the experiments imply that plasma protein(s) are required for the binding of pro-coagulant MVs to the bacteria and subsequent activation of clotting. Fibrinogen is a plausible candidate, as it is an abundant plasma protein and has high affinity for most streptococcal strains, including the AP1 strain, which was used in this study [9]. Therefore *S. pyogenes* bacteria were incubated with pro-coagulant MVs in the presence of normal or fibrinogen-depleted plasma, washed to remove non-bound MVs, and added to normal recalcified plasma. As before, when bacteria were pre-incubated with pro-coagulant MVs in the presence of normal plasma, clotting occurred within 169 s, while clotting was significant delayed (235 s) when bacteria were pre-incubated with pro-coagulant MVs in fibrinogen-depleted plasma, prior re-calcification with normal plasma (Figure 2B). Note that fibrinogen-depleted plasma was generated by defibrination and as fibrinogen was not completely removed (0,04 g/l are remaining), clotting was only delayed but not completely prevented.

Previous work has demonstrated that M1 protein from *S. pyogenes* is the main fibrinogen receptor on the AP1 strain used in this study [17]. To test whether M1 protein is also the major fibrinogen binding protein that mediates the interaction between bacteria and MVs, we employed an isogenic AP1 mutant strain (MC25), which does not express M1 protein on its surface [18]. Wildtype AP1 and MC25 bacteria were pre-incubated with pro-coagulant MVs in the presence of human plasma, washed thoroughly to remove non-bound MVs, and added to recalcified plasma. As depicted in figure 2C, MC25 bacteria tagged with pro-coagulant MVs were not as potent to induce clot formation as AP1 bacteria. The number of MV-bacterial aggregates was quantified by fluorescence microscopy and also in these experiments we found that the MC25 strain was not as effective as the AP1 strain to form aggregates (5±2 *vs.* 49±11) in plasma when opsonized with pro-coagulant MVs.

Finally we further investigated, whether other M proteins, either from the same serotype or from other serotypes, can recruit MVs to their surface. We therefore tested 14 clinical isolates, of which 5 were of the M1 type and 9 of other serotypes (Figure S2A and B). When subjecting these strains to clotting assays we found that all serotypes had similar pro-coagulant activities as seen for the AP1 strain.

Together the results show that the binding of pro-coagulant MVs to streptococci alters the bacterial surface from a non-coagulative to a pro-coagulative state. This interaction seems to be a common mechanism of group A streptococci, as also other serotypes explored similar clotting activities when incubated with pro-coagulant MVs. Moreover the data suggests that fibrinogen plays an important role in this chain of events.

### Pro-coagulant MVs recruit, via fibrinogen, streptococcal adhesion factors to their surface

**Figure 3:**
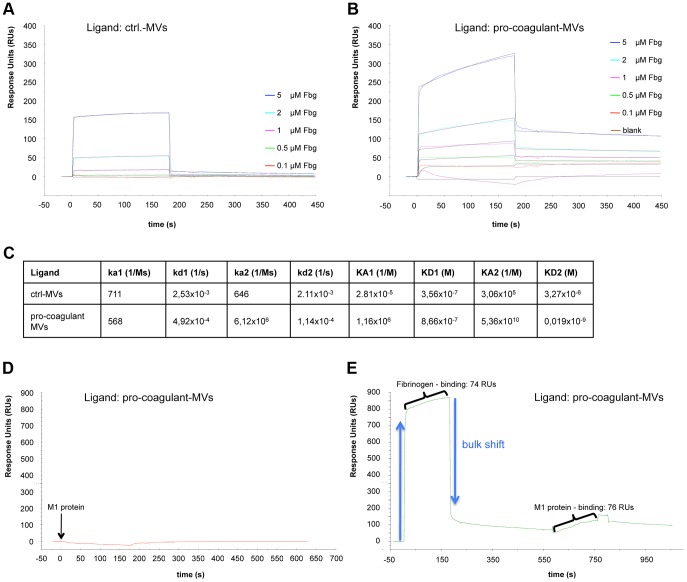
Binding properties of MV surfaces measured by SPR. Ctrl. MVs (**A**) or pro-coagulant MVs (**B**) were coupled to a sensor chip and subjected to injections with serial dilutions of fibrinogen (0.1–5 µM). Overlaid concentration-responses of the binding curves are shown. For both ligands, the data of the interaction with fibrinogen fit best to the heterogeneous binding model. The kinetic and affinity parameters are listed in (**C**). Pro-coagulant MVs were coupled to a sensor chip over which 100 µg/ml M1 protein was injected (**D**) or which was loaded sequentially, first with 5 µM fibrinogen, and then with 100 µg/ml M1 protein (**E**).

To study the role of fibrinogen as molecular bridge in more detail, surface plasmon resonance spectroscopy was employed. In a series of experiments we tested whether the activation state of MVs constitutes a regulatory mechanism that steers their affinity for fibrinogen. Sensor chips were coated with ctrl. or pro-coagulant MVs and probed with increasing concentration of fibrinogen. Though fibrinogen binding to both ctrl. MVs (Figure 3A) and pro-coagulant MVs (Figure 3B) was detected, determination of the association constants revealed that pro-coagulant MVs have a much higher affinity for fibrinogen than ctrl. MVs (0.019 nM vs. 3.3 µM, respectively) as shown in figure 3C.

The results from clotting experiments and fluorescent microscopy implicate an important role of M1 protein in binding pro-coagulant MVs (see Figure 2C). To verify this conclusion we measured the interaction between M1 protein and pro-coagulant MVs, immobilized on a sensor chip, by surface plasmon resonance in the presence or absence of fibrinogen. Figures 3D+E illustrates that an interaction between M1 protein and the pro-coagulant MVs was only detectable when the chip was pre-incubated with fibrinogen, confirming fibrinogen's function as a bridging factor. In conclusion, the data show that MVs derived from activated cells expose additional binding sites for fibrinogen, which are required as docking sites for the streptococcal adhesion factor such as M1 protein or M proteins from other serotypes.

### Fibrinogen-binding integrins are up-regulated on the surface of MVs

In order to investigate how pro-coagulant MVs can up-regulate additional fibrinogen binding-sites, mass spectrometry analysis was used, which allows the identification and quantification of intracellular, membrane associated, and secreted proteins of MVs. With this approach a total number of 169 proteins, with a false discovery rate of 1%, was identified in non-stimulated and pro-coagulant MVs (Table S1). In ctrl. MVs, 57% of the proteins were cytosolic, 23% secreted, 12% membrane-associated, and 8% mitochondrial origin (Figure S3). This composition changed drastically in pro-coagulant MVs, as here an increase in secreted and membrane associated proteins was found (36% and 28%, respectively), while a decrease in cytoplasm and mitochondrial proteins to 35% and 1% was measured (Figure S3). We also noted a rise in the concentration of 34 proteins recovered from pro-coagulant MVs comparing to ctrl. MVs (Table 2). In particular, leucocyte elastase levels were dramatically up-regulated (approximately 2500 times), but also higher levels of the fibrinogen-binding integrins CD18 (42 times) and CD11b (7.8 times) were noted. Another integrin, alpha-V/beta-3, which is a receptor for a number of human proteins including fibronectin, laminin, and vitronectin were also found upregulated (2.9 times). Finally we noticed that proteins with antimicrobial functions such as lysozyme and neutrophil defensin 1 (3.7 times and 2.8 times, respectively) were also enriched in pro-coagulant MVs. Taken together the determination of the protein content in ctrl. and pro-coagulant MVs by mass spectrometry analysis revealed that, apart from two fibrinogen-binding integrins, other proteins with an important role in the early immune response, are also up-regulated in pro-coagulant MVs.

**Table 2:** Proteins from pro-coagulant MVs detected by mass spectometry analysis that were significant upregulated, compared to ctrl. MVs [37].

*Proteins*	*pro-coagulant MVs*
Platelet glycoprotein Ib	1,505907916
Zyxin	1,519533276
CD36 antigen	1,630243882
Integrin alpha 2b , alpha2bbeta3, vibronectin	1,774382368
Myeloperoxidase	1,858537826
guanine nucleotide binding protein (G protein),	2,086581862
LIM and senescent cell antigen-like domains 1	2,158272675
Intercellular adhesion molecule 3	2,592230491
Monocyte differentiation antigen CD14	2,646502683
Platelet glycoprotein IX	2,805388155
Neutrophil defensin 1	2,855469867
Leukosialin	2,888946866
Integrin beta chain, alphaV, beta3 (Vitronectin receptor)	2,891676956
HLA class I histocompatibility antigen	2,897177578
Cytochrome b-245	3,055074965
Solute carrier family 9	3,07933824
Leukocyte common antigen (CD45 antigen)	3,242725034
CD44 antigen	3,455159577
Lysozyme C	3,743112539
Ras-related protein	3,827823588
Tetraspanin-14	4,135484955
Annexin I	4,533846343
SH3 domain-binding glutamic acid-rich-like protein 3	4,659452596
Integrin alpha-M precursor (CD11b)	7,840017118
Sodium/potassium-transporting ATPase alpha-1 chain	8,213665434
Annexin V	11,14117886
Brain abundant, membrane attached signal protein 1	12,4888186
Annexin A2	16,74723805
Annexin VI	18,68650302
Alanyl (Membrane) aminopeptidase (CD13)	32,75718975
Integrin, beta 2 (CD18)	42,36002149
Human Cofilin	74,66172249
Ras-related protein Rap-2a	98,69200927
Leukocyte elastase	2449,565037

### Plasma clots induced by pro-coagulant MVs immobilize bacteria

**Figure 4:**
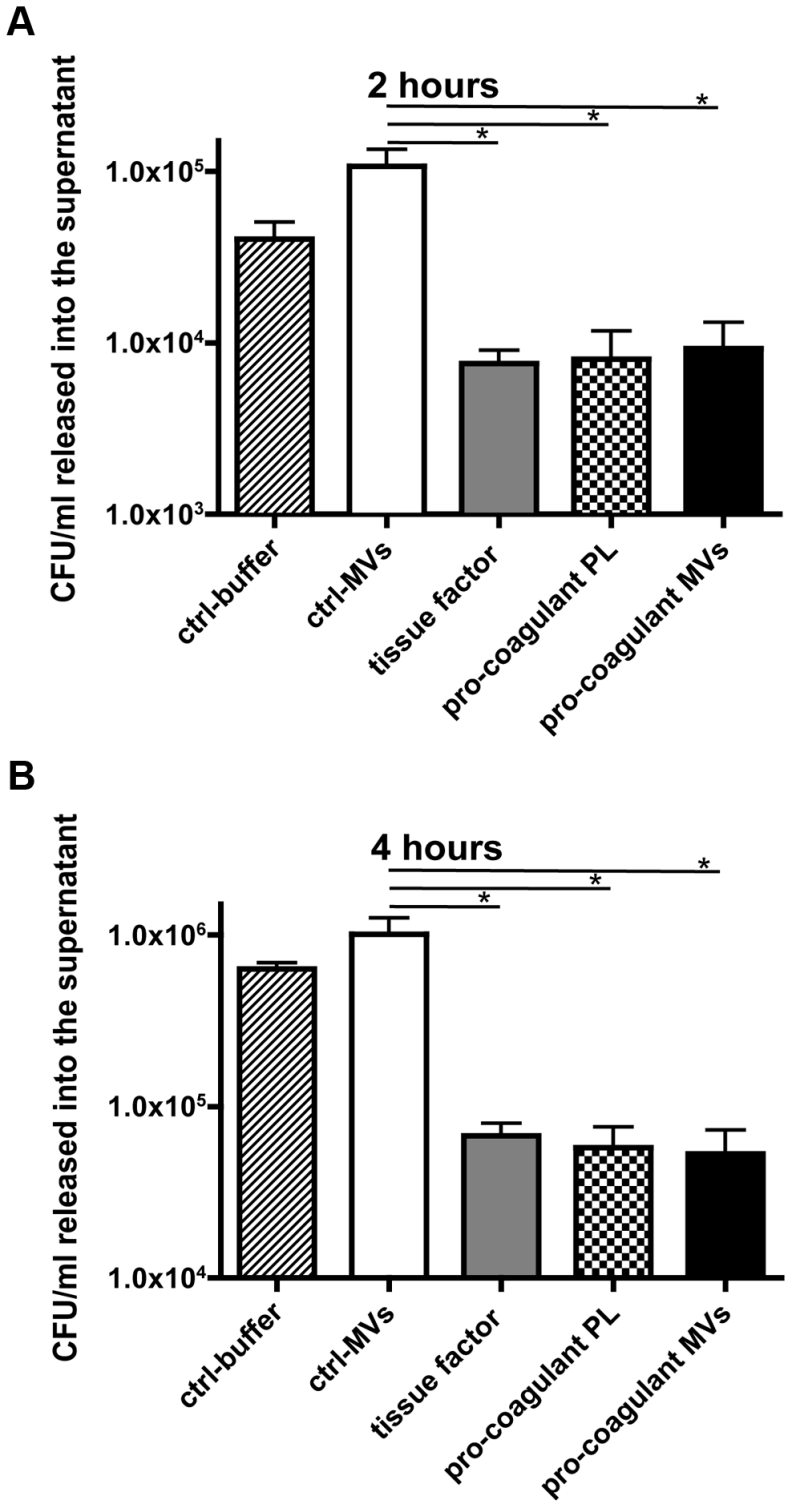
Pro-coagulant MV-derived clots prevent bacterial dissemination. Recalcified plasma was incubated with 2×10^5^ CFU/ml *S. pyogenes* and clot formation was triggered with pro-coagulant PLs or MVs or 2 pM tissue factor. Tris-buffer (ctrl-buffer) or ctrl-MVs samples were used as negative controls. The clots were covered with 1% plasma, incubated at 37°C and supernatants were plated after 2 (**A**) and 4 hours (**B**). The data represent the means ± SD of 3 independent experiments, *P<0.05.

Recent studies support the concept that clot formation at the site of infection entraps bacteria in the fibrin network, which in turn prevents bacterial spreading, and promotes bacterial elimination [19], [20]. Based on these reports, we speculated that MVs could also act as a clotting initiator that chains the bacteria within a formed clot. To prove this hypothesis, *S. pyogenes* were incubated in recalcified plasma followed by the addition of ctrl. MVs or pro-coagulant MVs. Artificial phospholipids with pro-coagulant activity (PLs) or tissue factor (TF) containing samples served as positive controls. Stable clots were formed when pro-coagulant MVs, PLs, or tissue factor were added to the bacteria/plasma mixture, while loose and less compact clots were generated when the bacteria/plasma mixture was incubated with buffer or ctrl. MVs (not shown). The clot samples were covered with Tris-buffer containing 1% plasma and incubated for two or four hours at 37°C. Aliquots were collected from the supernatants and bacterial loads were determined. After two hours of incubation the number of released bacteria from plasma clots derived by pro-coagulant MVs was significantly decreased (9.7 times), when compared with the number found in the supernatants of samples incubated with ctrl. MVs (Figure 4A). After the four-hour incubation, samples treated with buffer of ctrl. MVs contained high loads of streptococci. As seen before, incubation of bacteria in a plasma clot derived from pro-coagulant MVs prevented the escape of bacteria from the clots (more than 12 times, comparing to ctrl-MVs) and also PLs or tissue factor induced clots had a similar effect (Figure 4B). These data demonstrate that bacteria are efficiently trapped and immobilized if they are opsonized with pro-coagulant MVs.

### Plasma clots induced by pro-coagulant MVs have antibacterial activity

**Figure 5:**
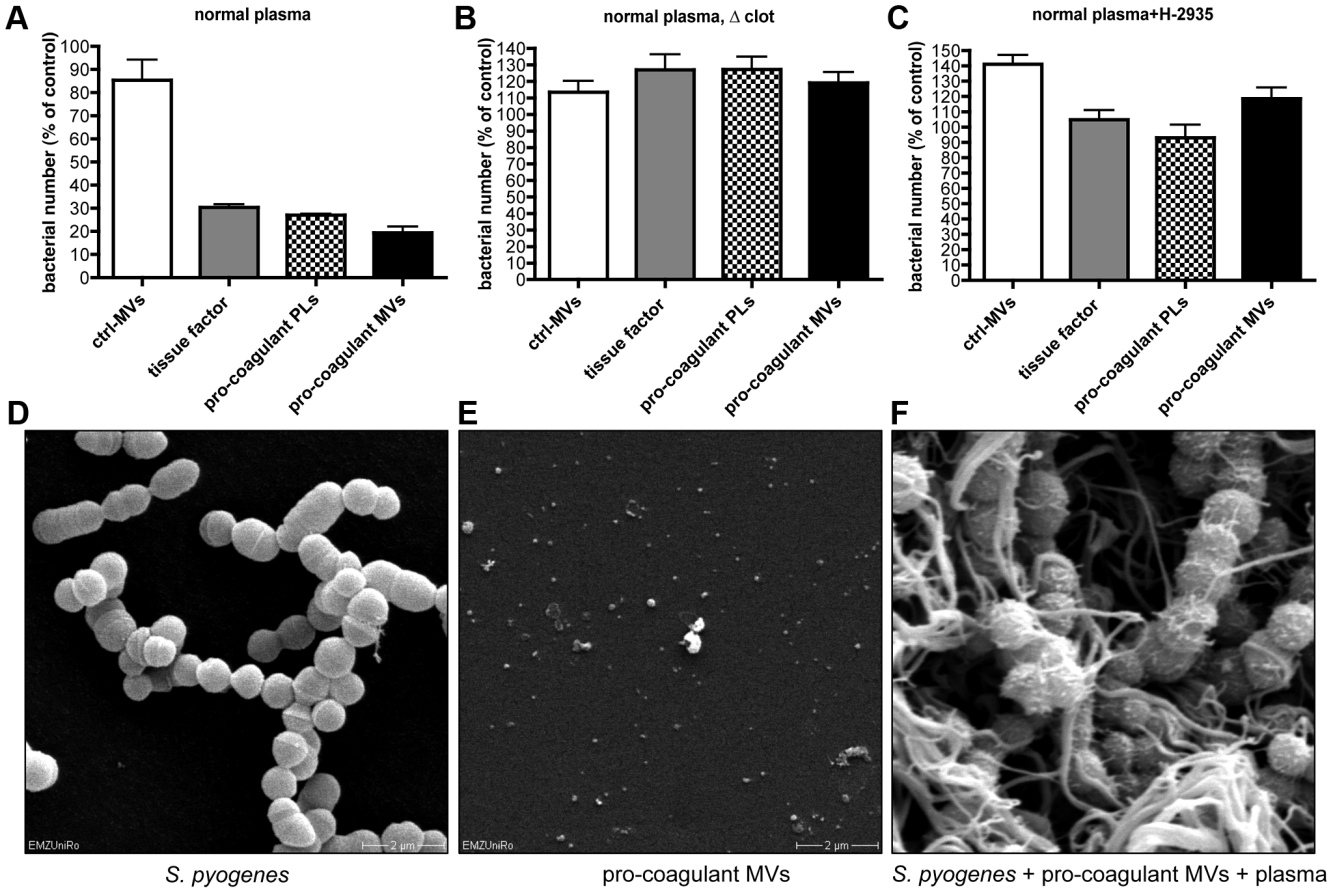
Determination of the antimicrobial activity of plasma clots induced by pro-coagulant MVs. **A**) Recalcified plasma was incubated with 2×10^5^ CFU/ml *S. pyogenes* in the presence of ctrl. MVs, pro-coagulant MVs, PLs, or 2 pM tissue factor. Tris-buffer was used as a control (100%). **B**) Samples were prepared as described in A, but clot formation was prevented by omitting calcium, or the addition of 1.5 mg/ml Gly-Pro-Arg-Pro (**C**). Scanning electron microscopy of *S. pyogenes* (**D**), pro-coagulant MVs (**E**), and a plasma clot with *S. pyogenes* that was induced by the addition of pro-coagulant MVs (**F**). The data represent the means ± SD of 3 independent experiments.

It has recently been shown that activation of the coagulation cascade on the surface of *S. pyogenes* leads to an induction of antimicrobial activity [20]. To investigate whether antimicrobial activity is also seen when clotting is induced by pro-coagulant MVs, additional bacterial growth experiments were performed. Streptococci were mixed with plasma and clotting was initiated by adding pro-coagulant MVs, PLs, or tissue factor. Ctrl. MVs or buffer served as controls. After 30 min, clots were homogenized and bacterial loads determined. As seen in figure 5A, bacterial counts were significant reduced to 20–30% in samples treated with pro-coagulant MVs, PLs, or tissue factor, when compared with ctrl. MVs. Samples incubated with buffer only, served as a control (100% growth). Clot formation appears to be the critical moment in these experiments, since no reduction in bacterial growth was monitored when calcium was omitted and thus clotting prevented (Figure 5B). Similar results, though not a complete reversion, were seen when recalcified samples were treated with a peptide (Gly-Pro-Arg-Pro) that prevents the polymerization of fibrin monomers (Figure 5C) [21].

To visualize the bacteria, samples were subjected to scanning electron microscopy (Figure 5D–F). In the absence of pro-coagulant MVs, *S. pyogenes* bacteria appear as intact cocci when incubated for 30 min in plasma (Figure 5D). In the next series of experiments, pro-coagulant MVs (Figure 5E) were added to the plasma bacteria mixture. Figure 5F illustrates that after activation with pro-coagulant MVs, bacteria were weaved in a fibrin network. It also appears that the morphology of bacteria was not significant compromised, as the bacterial cell membrane seems to be still intact (Figure 5F). These images may indicate that the effect seen is of bacteriostatic nature rather than bactericidal, however, more experimental support is needed to prove this conclusion. Taken together the data suggest that pro-coagulant MVs are able to prevent bacterial spreading and impair bacterial proliferation inside a plasma clot.

### Pro-coagulant MVs are increased in plasma samples from mice infected with *S. pyogenes*

**Figure 6:**
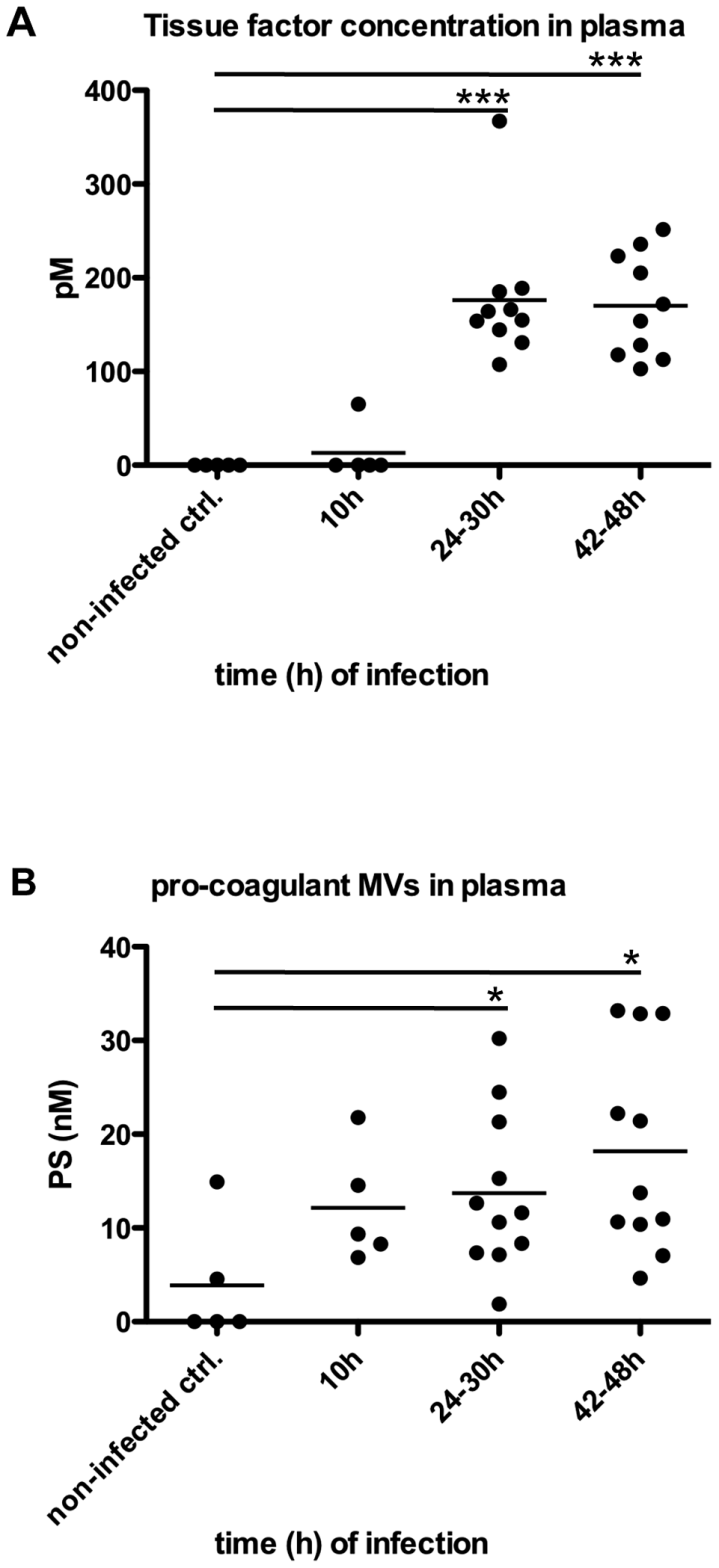
Analysis of plasma from septic mice. Mice were subcutaneously infected with 2×10^7^ CFU *S. pyogenes* bacteria and plasma samples were collected at 0, 24, 30, 42 and 48 hours after infection (n = 5–11/group). Tissue factor activity (**A**) and pro-coagulant MVs (**B**) expressed as PS equivalent (nM) were measured. *P<0.05, ***P<0.0001.

Recently we reported that pro-coagulant MVs from patients suffering from streptococcal sepsis are significant increased [13]. To test whether this can also be observed in an invasive animal model of streptococcal infection, mice were subcutaneously infected. The animals were sacrificed after three time points (10, 24–30, and 42–48 hours after infection) and plasma samples were recovered by cardiac puncture. Figure 6A depicts that the TF content in the plasma samples was not significant raised 10 hours after infection, but was significantly increased at the later time points (Figure 6A). Similar results were seen when measuring the concentrations of pro-coagulant MVs, though they already start to peak 10 hours after infection (Figure 6B). Thus, the data show that the generation of pro-coagulant MVs is part of the host response to invasive infection with *S. pyogenes*.

### Local treatment with pro-coagulant MVs dampens systemic bacterial spreading and improves survival in infected mice

**Figure 7:**
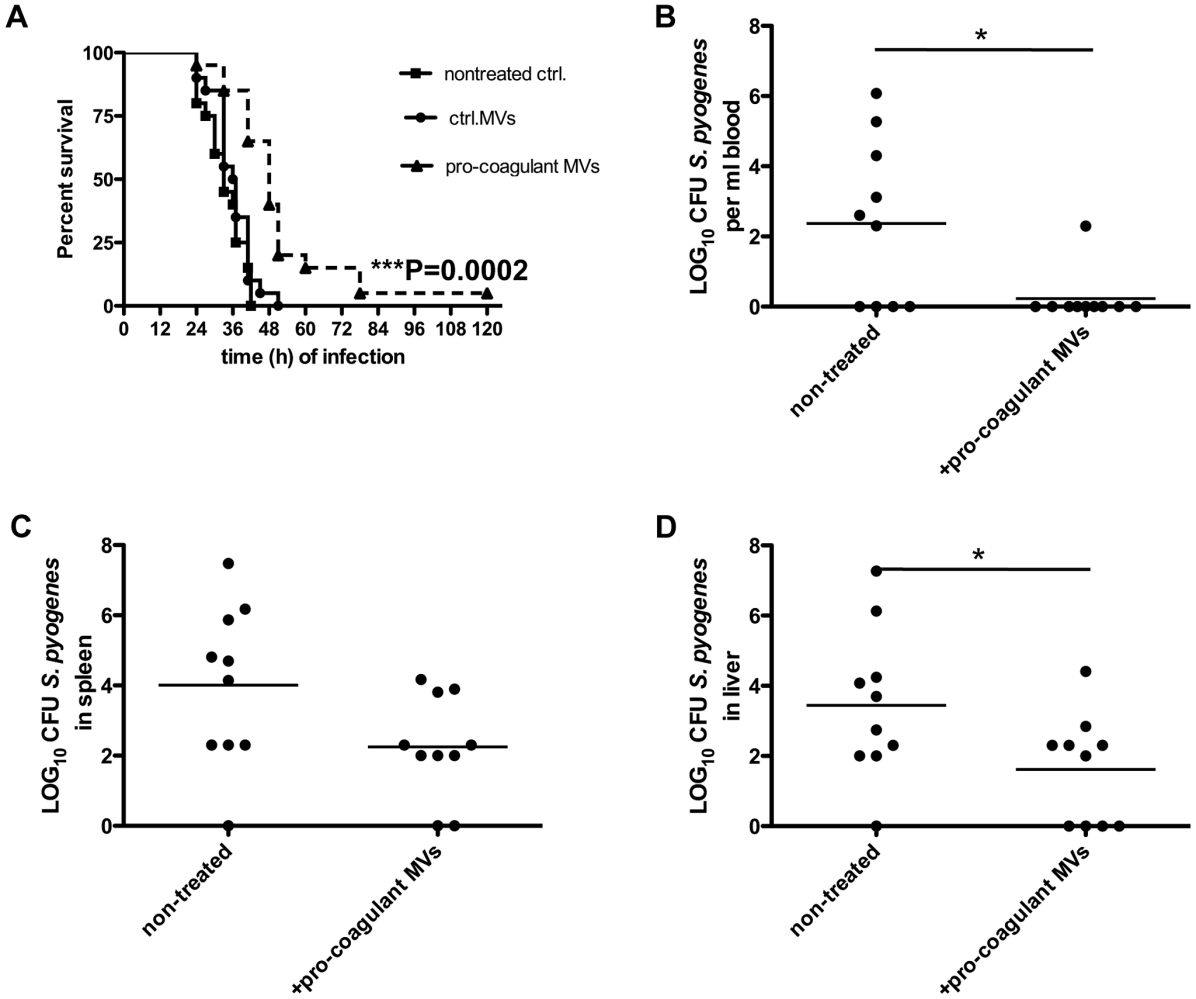
Treatment with pro-coagulant MVs in a mouse model of *S.* *pyogenes* sepsis dampens bacterial dissemination and improves survival. **A**) Mice were subcutaneously infected with 2×10^7^ CFU *S. pyogenes* bacteria, immediately treated with 100 µl ctrl. or pro-coagulant MVs (150 MPs/µl, derived from 2×10^6^ PBMCs) and survival was monitored up to 120 h after infection. Results show 4 independent experiments with MVs from 4 different donors and a total amount of 20 animals per group. (**B–D**) Mice were infected as described above and were immediately treated with 100 µl pro-coagulant MVs (150 MPs/µl, derived from 2×10^6^ PBMCs), non-treated mice served as controls. Eighteen hours after infection, mice were killed and bacterial loads in the blood (**B**), spleen (**C**), and liver (**D**) were determined. Data are presented as means of 10 mice per group and were obtained from 2 independent experiments (*P<0.05).

The role of MVs in systemic infectious diseases is currently not completely understood, but it has been speculated that elevated levels in the early phase of sepsis may have protective effects [22]. We therefore studied whether the local application of pro-coagulant MVs to the site of infection may improve the outcome of the disease. Three groups of mice were infected with *S. pyogenes* bacteria and were treated either with vehicle, ctrl. MVs or pro-coagulant MVs. While application of ctrl. MVs failed to improve survival as compared to control (vehicle), treatment with pro-coagulant MVs significant prolonged survival time and decreased the mortality rate (Figure 7A).

The subcutaneous injection of pro-coagulant MVs also had an impact on the bacterial load in different organs of the infected mice. Mice received a subcutaneous injection of *S. pyogenes* bacteria and simultaneously a single dose of pro-coagulant MVs. Infected animals were sacrificed 18 hours after infection, and bacterial loads in the blood, liver, and spleen were determined. As depicted in figure 7B–D, treatment with pro-coagulant MVs resulted in decreased numbers of bacteria in all organs when compared with non-treated animals. These results are in line with previous conclusions and may indicate that pro-coagulant MVs are part of the early host defense to an infection at an early stage of the infectious disease progression.

## Discussion

Pro-coagulant MVs constitute one of the main reservoirs of blood-borne TF, which are released from monocytes, macrophages, or endothelial cells with inducible TF expression [23] and they are therefore considered to be key determinants of the hemostasis equilibration [24]. Notably, the number of pro-coagulant MVs can significantly increase in patients suffering from sepsis as reported by us and other groups [13], [25]. These findings raise the question whether they are part of the host response to infection or rather contribute to systemic hemorrhagic complications, such as disseminated intravascular coagulation (DIC) in severely ill patients. Reid and Webster recently published a review article on the role of MVs in sepsis [22]. The authors conclude that MVs are beneficial at the early stage of sepsis as they can compensate for some of the host's systemic reactions [22]. Our findings support this notion, because local treatment with pro-coagulant MVs significantly prevents bacterial dissemination and improves survival. Moreover, activation of PMBCs is triggered by the binding of M1 protein to toll-like receptor 2 [12], which suggests that formation and release of pro-coagulant MVs follows the principles of pattern recognition and are therefore part of the innate immune reaction. However, as seen for many other host defense mechanisms, the systemic induction of pro-coagulant MVs may contribute to severe complications, such as DIC. A better understanding of the molecular mechanisms that modulates the tightly regulated process may lead to the development of novel antimicrobial therapies with different modes of action that can be used for local treatment or in systemic complications.

Microvascular thrombosis and the formation of a fibrin network can be considered as an efficient and early response of the host defense against bacteria that can lead to an immobilization of bacteria and thereby attenuates the spreading of the pathogen [19], [20], [26]. Our studies show that pro-coagulant MVs bind to *S. pyogenes* and that this interaction leads to an alteration of the bacterial surface into a pro-coagulative state. We found that fibrinogen is a docking molecule that attaches protein M1, a streptococcal surface-bound adhesion factor, to pro-coagulant MVs. Subsequent mass spectroscopic analysis revealed an up-regulation of the fibrinogen binding integrins (CD18 and CD11b, respectively) at the surface of pro-coagulant MVs. This chain of events presents a plausible explanation as to how pro-coagulative MVs achieve their affinity for *S. pyogenes*. However, it cannot be ruled out that other proteins such as fibronectin, vitronectin or laminin are also involved [27]–[29]. Notably, many of these host adhesion factors are also receptors for other bacterial pathogens [30]. Thus their binding to pro-coagulant MVs may represent some kind of pattern recognition mechanism that allows the targeting of other microorganisms in a more general sense. Future work will show whether pro-coagulant MVs are also interacting with other bacterial species and whether this involves the recruitment of fibrinogen and/or other host adhesion proteins.

Our results show that plasma clots that were induced by pro-coagulant MVs can immobilize *S. pyogenes* as efficiently as clots induced by tissue factor or artificial phospholipids. Importantly, clots formed in the presence of pro-coagulant MVs had antimicrobial activity against *S. pyogenes*, which could be explained by their cargo containing antimicrobial peptides and proteins. However, we noted that clots formed by the addition of tissue factor or artificial phospholipids, were also able to kill the entrapped bacteria. It therefore remains to be determined as to what extent the peptides/proteins with antimicrobial activity from pro-coagulant MVs contribute to bacterial killing, or if there are bactericidal substances generated during the activation of the coagulation cascade. The latter hypothesis is supported by recent findings that many coagulation factors contain a sequence at their carboxy-terminal part with an antimicrobial activity [31], [32]. Taken together, our data show that activation of the coagulation cascade and the formation of a fibrin network are important mechanisms to prevent bacterial dissemination and proliferation. As pro-coagulant MVs are induced at an early stage during bacterial infection, their local interaction with bacteria can be considered as part of the early immune response.

## Material and Methods

### Bacterial strains and culture conditions

The *S. pyogenes* strain AP1 (40/58) serotype M1 and its M1-derivate MC25 have been described previously [18], [33]. All other *S. pyogenes* strains were clinical isolates from our strain collection that have been characterized by standard microbiological procedures. Bacteria were grown overnight in Todd-Hewitt broth (THB; GIBCO) at 37°C and 5% CO_2_.

### Material

M1 protein was purified from the supernatant of *S. pyogenes* MC25, as previously described [18]. Artificial pro-coagulant phospholipids were from Rossix (Sweden). Recombinant tissue factor and anti TF were from American Diagnostica (Germany). Anti CD14 was from Dako (Denmark).

### Preparation of MVs from peripheral blood mononuclear cells (PBMCs)

PBMC isolation, stimulation as well as MV purification were performed as described previously and used at concentration range from 50 to 150 MPs/µl [13].

### Clotting experiments

*S. pyogenes* bacteria from 10 ml overnight culture were washed and resuspended in 1 ml 10 mM HEPES-buffer (2×10^9^ CFU/ml). 150 µl bacteria and 30 µl MVs - in the presence or absence of 300 µl human plasma – were mixed and incubated for 30 min. at 37°C. Alternatively, fibrinogen depleted plasma (Affinity Biologicals, Canada) was used. Bacteria were washed 3 times with HEPES-buffer by centrifugation (1550× g for 10 min.), and finally resolved in HEPES-buffer. Clotting time was measured in a coagulometer (Amelung) after addition of reaction mixtures to recalcified normal human plasma.

### Bacterial immobilization in plasma clots

100 µl recalcified normal plasma was mixed with 25 µl 2×10^5^ CFU *S. pyogenes* bacteria, 25 µl MVs or pro-coagulant PLs (0.25 mM, Rossix), or 2 pM tissue factor (American Diagnostica) and incubated at 37°C for 5 min. The clots were covered with 10 mM Tris-buffer containing 1% plasma. At the indicated time points, 100 µl aliquots of the supernatant were plated onto blood agar in 10-fold serial dilutions and the number of bacteria was determined by counting colonies after 18 hours of incubation at 37°C.

### Antimicrobial activity of plasma clots

Plasma clots were produced as described above, covered with Tris-buffer containing 1% plasma and incubated at 37°C for 30 min. Alternatively, the tetrapeptide Gly-Pro-Arg-Pro (Bachem) was added to prevent clotting (1.5 mg/mL final concentration). After incubation clots were disrupted in a Ribolyser (Hybaid, 30 sec at speed 4.0) and the homogenate was plated directly onto blood agar. The number of bacteria was determined by counting colonies after 18 hours of incubation at 37°C.

### Fluorescence microscopy

MVs were labeled with the red fluorescence aliphatic chromophore PKH26 dye (Sigma), which intercalate into lipid bilayers [34]. After labeling, MVs were washed and centrifuged as described [13]. 150 µl bacteria (2×10^9^ CFU/ml) and 30 µl labeled MVs were mixed in 300 µl human plasma and incubated for 30 min at 37°C. After incubation 10 µl of the mix was dropped onto a cover slide, counterstained with DAPI (Invitrogen) and visualised by a BX60 fluorescence microscope and 100×1.3 or 60×1.25 UplanFl objectives (Olympus, Hamburg, Germany).

### Negative staining and transmission electron microscopy

Human proteins (annexin V, anti CD14 AB, and anti TF AB) were labeled with colloidal gold (15 and 5 nm in diameter, BBI International) as described earlier [35]. MV/*S. pyogenes* preparations were mixed with gold-labeled 20 nM proteins for 20 min at room temperature and processed for negative staining [36].

### Scanning Electron Microscopy (SEM)

Clots were fixed with 2.5% glutaraldehyde overnight. Samples were washed 2–3 times with 0.1 M sodium phosphate buffer (pH 7.3), dehydrated with a series of increasing ethanol concentrations (5 minutes in 30%, 5 minutes in 50%, 10 minutes in 70%, 10 minutes in 90% and two times 10 minutes in ethanol absolute), and dried with CO_2_ by critical point method with a Emitech dryer as outlined by the manufacturer. Dried samples were covered with gold to a 10 nm layer and scanned with a Zeiss DSM 960A electron microscope.

### TF activity ELISA

The Actichrome TF activity assay kit (American Diagnostica) was used to quantify the TF pro-coagulant activity in the plasma samples [13].

### Measurement of MP pro-coagulant activity

The Coa-MP activity kit (Coachrom Diagnostica) was used according to the instructions of the manufactory, to measure the pro-coagulant activity of MVs in plasma [13].

### Proteomic analysis

Protein digestion was carried out as previously described [37]. The resulting peptide mixtures were concentrated using spin-columns from Harvard Apparatus using the manufactures' instructions.

The hybrid Orbitrap-LTQ XL mass spectrometer (Thermo Electron, Bremen, Germany) was coupled online to a split-less Eksigent 2D NanoLC system (Eksigent technologies, Dublin, CA, USA). Peptides were loaded with a constant flow rate of 10 µl/min onto a pre-column (Zorbax 300SB-C18 5×0.3 mm, 5 µm, Agilent technologies, Wilmington, DE, USA) and subsequently separated on a RP-LC analytical column (Zorbax 300SB-C18 150 mm×75 µm, 3.5 µm, Agilent technologies) with a flow rate of 350 nl/min. The peptides were eluted with a linear gradient from 95% solvent A (0.1% formic acid in water) and 5% solvent B (0.1% formic acid in acetonitrile) to 40% solvent B over 55 minutes. The mass spectrometer was operated in the data-dependent mode to automatically switch between Orbitrap-MS (from m/z 400 to 2000) and LTQ-MS/MS acquisition. Four MS/MS spectra were acquired in the linear ion trap per each FT-MS scan which was acquired at 60,000 FWHM nominal resolution settings using the lock mass option (m/z 445.120025) for internal calibration. The dynamic exclusion list was restricted to 500 entries using a repeat count of two with a repeat duration of 20 seconds and with a maximum retention period of 120 seconds. Precursor ion charge state screening was enabled to select for ions with at least two charges and rejecting ions with undetermined charge state. The normalized collision energy was set to 30%, and one microscan was acquired for each spectrum.

The data analysis was performed as previously described [37]. Briefly, the MS2 spectra were searched through the X! Tandem 2008-05-26 search engine [38] against the human protein database. The search was performed with semi-tryptic cleavage, specificity, 1 missed cleavages, mass tolerance of 25 ppm for the precursor ions and 0.5 Da for fragment ions, methionine oxidation as variable modification and cysteine carbamidomethylation as fixed modification. The database search results were further processed using the Trans-Proteomic pipeline, version 4.4.0 [39].

### Surface plasmon resonance binding study

Real time biomolecular interaction was analyzed with a BIAcore3000 system (Biosensor, La Jolla, CA) using L1 sensor chips [40]. The L1 sensor chip comprises a carboxymethyl dextran hydrogel derivatized with lipophilic alkyl chain anchors to capture phospholipid vesicles. Experiments were performed at 25°C with 10 mM TRIS, 0.9% NaCl, pH 7.4 as running buffer and PBS (pH 7.4) as immobilization buffer. MVs were coated onto the L1-sensor chip according to the manufacturer' s instructions. Briefly, the L1 chip surface was washed by 2×3 minute injections of 40 mM N-octyl-β-D-glucopyranoside (Roth, Germany) at a flow-rate of 10 µl/min. MVs in PBS were then injected over the sensor for 30 min at a flow-rate of 2 µl/min resulting in 2000–2200 response units (RUs) of ctrl or pro-coagulant MVs. To remove residual multilayer structures and loosely bound vesicles, a short pulse of 10 mM NaOH was applied. Subsequently, BSA (0.1 mg/ml, 5 min) was added to block non-specific surface binding. The resulting bilayer linked to the chip surface was taken as a model MV-membrane surface for studying fibrinogen binding. Pure buffer solutions and the solution containing fibrinogen or M1 protein were applied at a flow rate of 10 µl/min. Following each cycle of analysis, the sensorchip was regenerated either with short pulses of 10 mM NaOH leaving the MV-lipid monolayer intact for additional interaction studies, or with 40 mM N-octyl-β-D-glucopyranoside stripping the MV-lipid layer from the surface in order to adsorb new MVs.

### SPR data analysis

The sensorgrams for each fibrinogen–MV bilayer interaction were analyzed by curve-fitting. Data from 5 concentrations were selected for statistic analysis. RUs of the running buffer were subtracted from the RUs of the sample solution. The data were analyzed using the Biaevaluation 3.0 software (Biacore) that offers various reaction models to perform complete kinetic analyses. The data from the BIAcore sensorgrams were fitted globally, and the heterogeneous ligand model resulted in optimum mathematical fits, reflected by low χ^2^ values (<5).

### Animal experiments

The subcutaneous infection model with *S. pyogenes* AP1 strain were performed in female Balb/C mice as described previously [33]. This study was performed in strict accordance with the recommendations in the Guide for the Care and Use of Laboratory Animals of the National Institutes of Health. The protocol was approved by the Committee on the Ethics of Animal Experiments the *Landesveterinär- und Lebensmitteluntersuchungsamt Rostock* (Permit Number: *7221.3-1.1-031/10.*).

### Statistical analysis

Statistical analysis was performed using GraphPad Prism, Version 4.00. The P-value was determined by using the unpaired t-test (comparison of 2 groups) or the log-rank test (comparison of survival curves). All samples were analyzed in triplicate and all experiments were performed at least three times, if not otherwise declared. The bars in the figures indicate standard deviation.

## Supporting Information

Figure S1 **Representative transmission fluorescence microscopy images (from 3 experiments) from** ***S. pyogenes*** **incubated with MVs in plasma.** 150 µl bacteria (2×10^9^ CFU/ml, blue) and 30 µl PKH-26 labeled MVs (red) were mixed in 300 µl human plasma and incubated for 30 min at 37°C. After incubation 10 µl of the mix were dropped on a coverslide, counterstained with DAPI (blue, Invitrogen) and investigated. Scale bars represent 10 µm.
(TIF)

Figure S2 **Clotting of different M1 (A) or M protein (B)** ***S. pyogenes*** **strains after incubation with pro-coagulant MVs.** Bacteria were incubated with pro-coagulant MVs in the presence of plasma for 30 min at 37°C. After washing, bacteria or buffer (buffer-ctrl.) were added to recalcified plasma and clotting time was determined. Clotting times were performed in triplicate. The data represent the means ± SD of 2 independent experiments.
(TIF)

Figure S3 **Subcellular location of proteins from PBMC derived MVs identified by hybrid orbitrap mass spectrometry analysis.**
(TIF)

Table S1 **Result of proteomic analysis of ctrl-MVs and pro-coagulant MVs from PBMCs.** All identified proteins are listed, with a false discovery rate of 1%.
(XLSX)
