# Supplementary figures and images for: A Novel Role for Pro-Coagulant Microvesicles in the Early Host Defense against Streptococcus pyogenes
Source: PLoS Pathog. 2013 Aug 1;9(8):e1003529. doi: 10.1371/journal.ppat.1003529 (PMC3731245; doi:10.1371/journal.ppat.1003529)

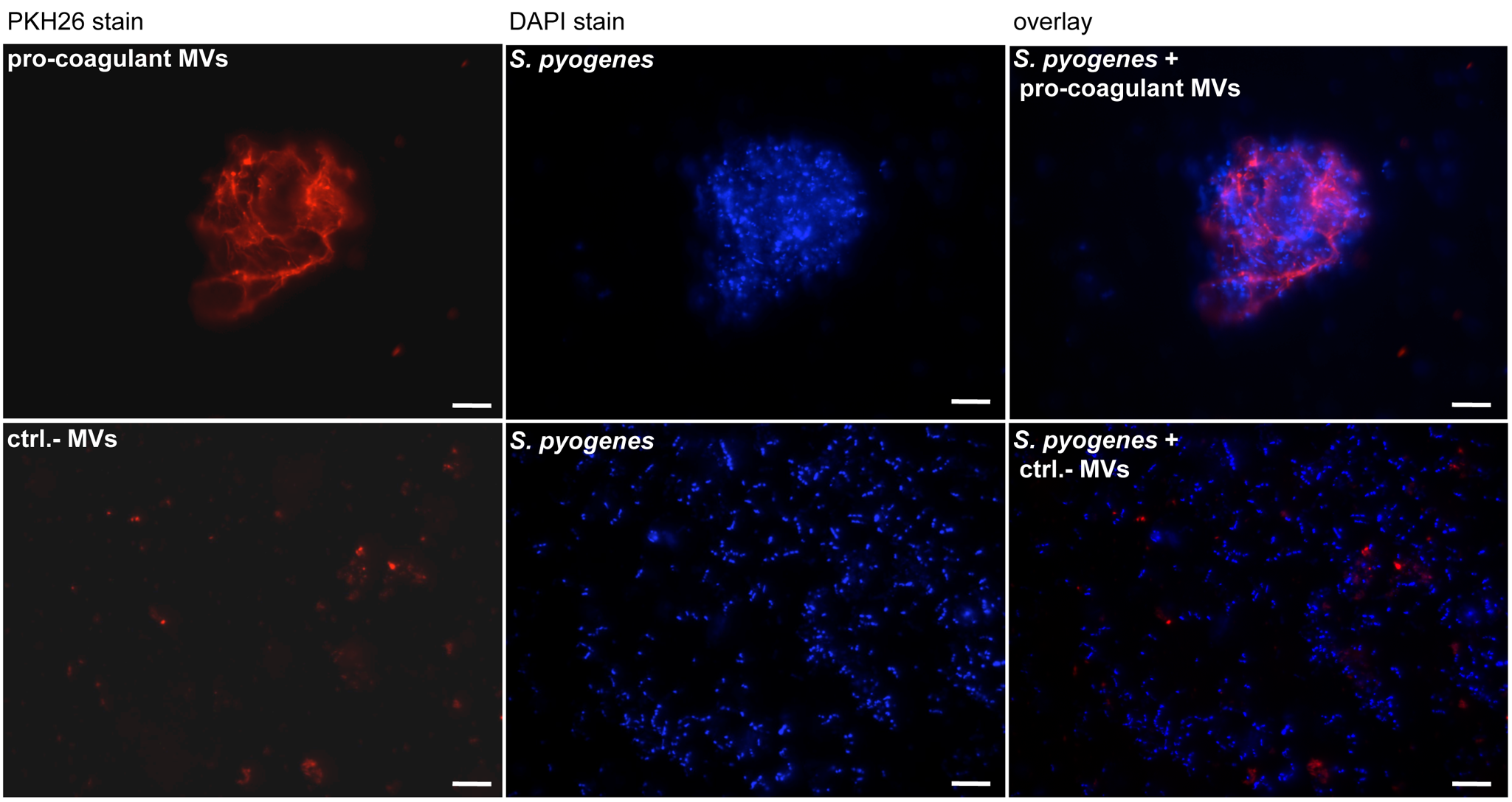

Supplement: Figure S1 — Representative transmission fluorescence microscopy images (from 3 experiments) from S. pyogenes incubated with MVs in plasma. 150 µl bacteria (2×10^9^ CFU/ml, blue) and 30 µl PKH-26 labeled MVs (red) were mixed in 300 µl human plasma and incubated for 30 min at 37°C. After incubation 10 µl of the mix were dropped on a coverslide, counterstained with DAPI (blue, Invitrogen) and investigated. Scale bars represent 10 µm. (TIF) [file ppat.1003529.s001.tif]

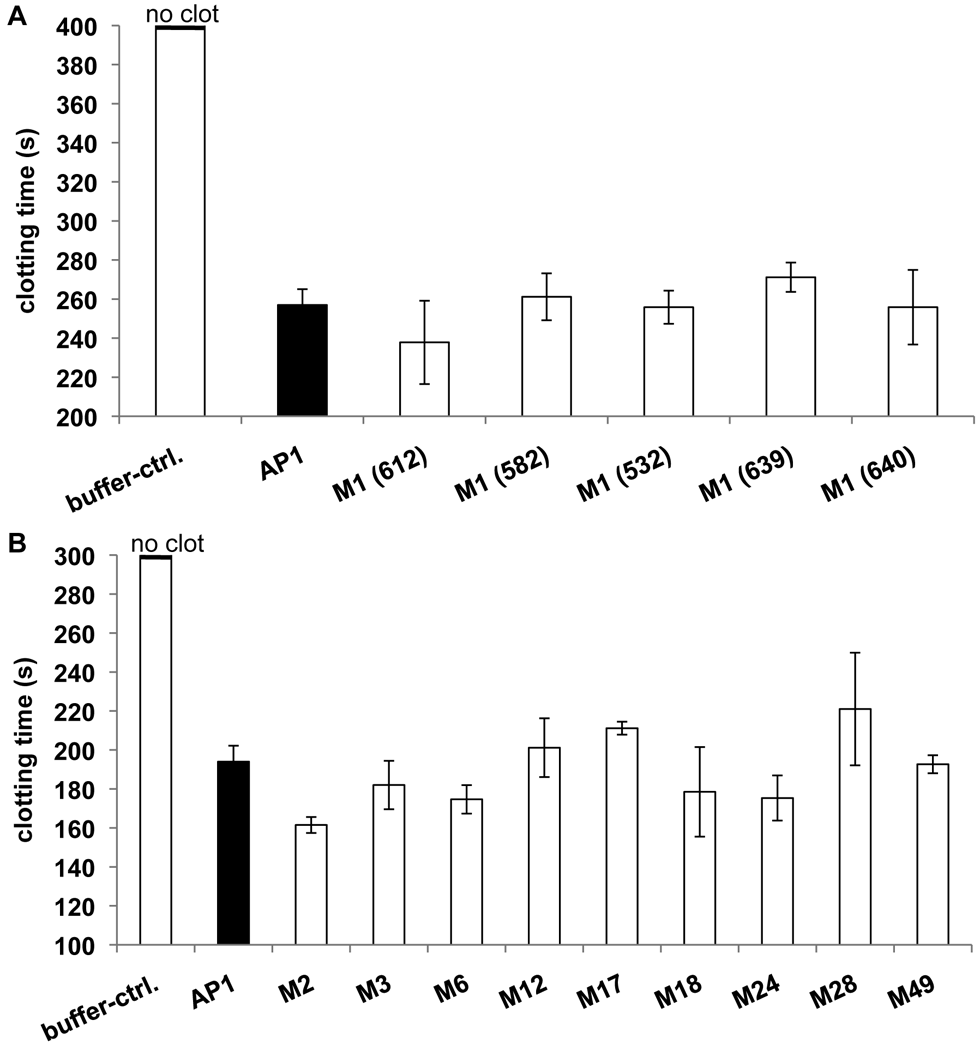

Supplement: Figure S2 — Clotting of different M1 (A) or M protein (B) S. pyogenes strains after incubation with pro-coagulant MVs. Bacteria were incubated with pro-coagulant MVs in the presence of plasma for 30 min at 37°C. After washing, bacteria or buffer (buffer-ctrl.) were added to recalcified plasma and clotting time was determined. Clotting times were performed in triplicate. The data represent the means ± SD of 2 independent experiments. (TIF) [file ppat.1003529.s002.tif]

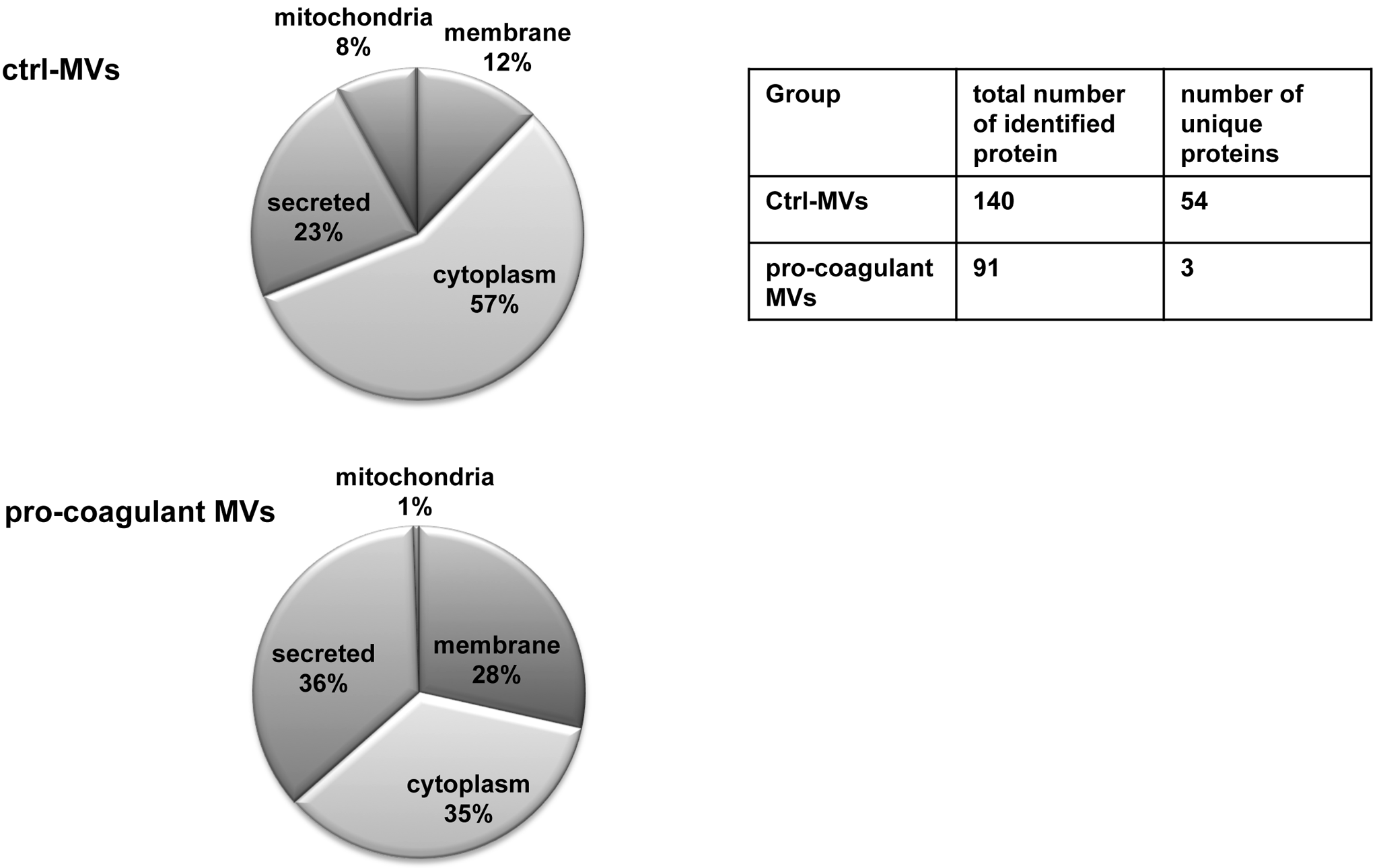

Supplement: Figure S3 — Subcellular location of proteins from PBMC derived MVs identified by hybrid orbitrap mass spectrometry analysis. (TIF) [file ppat.1003529.s003.tif]
